# MYC regulates the non-coding transcriptome

**DOI:** 10.18632/oncotarget.3033

**Published:** 2014-12-30

**Authors:** Jonathan R. Hart, Thomas C. Roberts, Marc S. Weinberg, Kevin V. Morris, Peter K. Vogt

**Affiliations:** ^1^ Department of Molecular and Experimental Medicine, The Scripps Research Institute, La Jolla, CA, USA; ^2^ Department of Physiology, Anatomy and Genetics, University of Oxford, Oxford, United Kingdom; ^3^ Antiviral Gene Therapy Research Unit, Department of Molecular Medicine and Haematology, School of Pathology, University of the Witwatersrand, WITS, South Africa; ^4^ School of Biotechnology and Biomedical Sciences, University of New South Wales, NSW, Australia

**Keywords:** Transcriptional regulation, RNA-seq, promoter occupancy, bidirectional promoter, nuclear run-on, quantitative PCR

## Abstract

Using RNA-seq (RNA sequencing) of ribosome-depleted RNA, we have identified 1,273 lncRNAs (long non-coding RNAs) in P493-6 human B-cells. Of these, 534 are either up- or downregulated in response to MYC overexpression. An increase in MYC occupancy near their TSS (transcription start sites) was observed for MYC-responsive lncRNAs suggesting these are direct MYC targets. MYC binds to the same TSS across several cell lines, but the number of TSS bound depends on cellular MYC levels and increases with higher MYC concentrations. Despite this concordance in promoter binding, a majority of expressed lncRNAs are specific for one cell line, suggesting a determinant role of additional, possibly differentiation-specific factors in the activity of MYC-bound lncRNA promoters. A significant fraction of the lncRNA transcripts lack polyadenylation. The RNA-seq data were confirmed on eight selected lncRNAs by NRO (nuclear run-on) and RT-qPCR (quantitative reverse transcription PCR).

## INTRODUCTION

MYC is a basic helix-loop-helix leucine zipper (bHLHLZ) protein that controls cell proliferation, differentiation, metabolism, apoptosis, and the maintenance of pluripotency. It is a key component of a broad transcription factor network, forming heterodimers with the bHLHLZ protein MAX [1-4]. MYC-MAX dimers bind to DNA Enhancer (E)-box elements with the consensus sequence CACGTG at target gene promoters and positively or negatively regulate gene expression [5].

MYC plays a critical role in human cancer [3, 6]. In numerous tumor types, it shows gain of function, primarily through overexpression or amplification [7, 8]. The classic example is Burkitt's lymphoma in which a t(8;14) translocation brings the transcription of *MYC* under the control of the immunoglobulin heavy chain locus [9], leading to ectopically high levels of MYC expression. In other tumor types, the incidence of MYC gain of function extends from 5 to 45% [7, 8]. The traditional view of MYC-driven tumorigenesis is that the MYC protein behaves as a classical transcription factor, which regulates the expression of a specific set of downstream genes that contribute to cancer progression. However, it has been shown recently that MYC-mediated transcriptional regulation is so widespread that MYC can be considered an ‘amplifier’ of transcription on a global scale [10-13].

Studies on MYC-mediated transcriptional regulation have focused mainly on coding transcripts; specific links between MYC and non-coding RNAs (ncRNAs) have been investigated only recently [14-19]. The importance of ncRNAs, such as transfer RNAs, ribosomal RNAs and spliceosome-associated RNAs, has been recognized since the formative years of molecular biology. However, the recent development of high-throughput sequencing and RNA tiling array technologies has revealed the full extent to which mammalian genomes are pervasively transcribed [20, 21]. It is now known that the majority of gene loci produce multiple interlaced [22] and overlapping transcripts [23] in both sense and antisense orientations [24, 25]. Of particular interest are the long non-coding RNAs (lncRNAs). lncRNAs are mRNA-like transcripts (>200 nucleotides) with low protein coding potential. A variety of diverse functions have been ascribed to lncRNAs including post-transcriptional gene silencing [26-29], epigenetic regulation [30-34], modulation of transcription factor function [35], modulation of alternative splicing [36, 37], nuclear organization [38-40], sub-cellular trafficking [41], generation of small RNAs [42-44], sequestration of signaling proteins [45], telomere function [46], and regulation of 3-dimensional chromatin structure [47]. lncRNAs are therefore important components of the gene regulatory apparatus of the cell. Yet the control and functional mechanisms of most lncRNAs are only just beginning to be explored [48]. Here we show that the broad transcriptional control that MYC exerts on the coding transcriptome also extends to the lncRNA transcriptome.

## RESULTS

### Numerous lncRNAs are regulated by MYC in P493-6 cells

**Figure 1:**
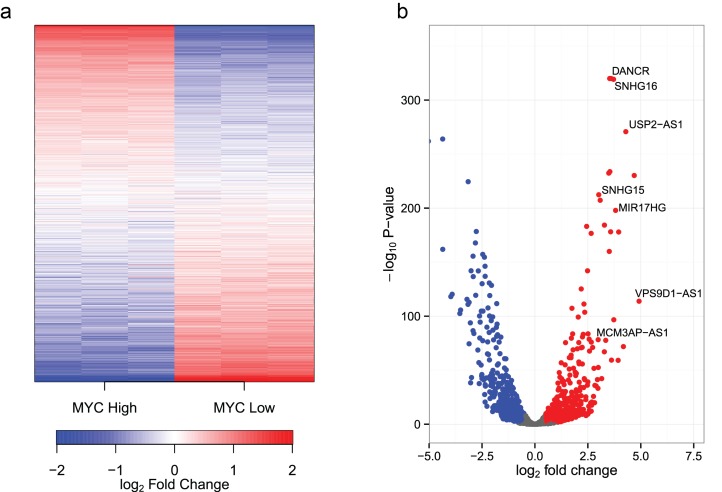
MYC regulates the expression of ncRNAs (a) A heatmap of ncRNAs detected in P493-6 cells. P493-6 cells were analyzed by RNA-seq in triplicate under conditions of high and low MYC expression. Transcripts are ordered by the change in expression upon MYC upregulation with those most upregulated at the top and those most downregulated at the bottom. $\log_{2}FC_{i,\mathrm{gene}}\;=\;\log_{2}CPM_{i,\mathrm{gene}}\;-\;\frac{1}{N}\sum_{i=1}^{N}\log_{2}CPM_{i,\mathrm{gene}}$ where i refers to the sample (column), gene refers to the gene (row) and N refers to the total number of samples (6). (b) A volcano plot of observed fold changes in ncRNA expression vs significance.

**Figure 2:**
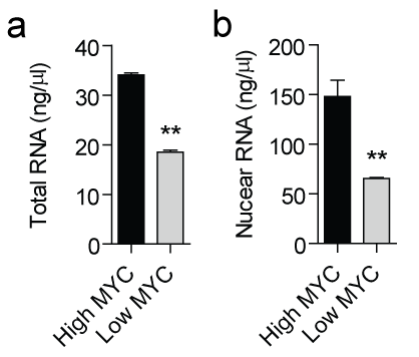
Total and nuclear RNA content at high and low MYC levels Total RNA was prepared using an RNA extraction robot from (a) ~2×10^5^ P493-6 cells with high or low expression of MYC, or (b) ~4×10^6^ purified nuclei in triplicate. Samples were eluted in a fixed volume of water and RNA concentration determined by Nanodrop. Values are mean + SEM, **P<0.01.

To study the effect of MYC on the non-coding transcriptome, we used the human B-cell line P493-6. P493-6 cells express high levels of MYC from an integrated transgene under the control of a tet-off promoter [49]. These cells are therefore a useful system for studying MYC target genes, as the addition of doxycycline reduces MYC expression by >40 fold. To investigate the possible role of MYC in regulating the non-coding RNA transcriptome, triplicate cultures of P493-6 cells were prepared in the presence (low, endogenous MYC) or absence (high MYC) of doxycycline. Total RNA was harvested after 24 hours and transcript expression levels determined by RNA sequencing (RNA-seq). Total RNA samples were depleted of ribosomal RNAs and strand-specific cDNA libraries were prepared in order to maximize sequencing depth, distinguish between sense and antisense RNAs, and to enable the measurement of transcripts irrespective of their 3′ terminal polyadenylation status. Raw reads were aligned to the hg19 build of the human genome, and reads uniquely aligning with non-coding transcripts (as defined in the Gencode v19 non-coding transcripts [50, 51]) were counted [52]. A total of 1,273 non-coding transcripts were detected (Supplemental Table 1). MYC significantly upregulated 296 and downregulated 238 non-coding transcripts by more than 2-fold (*P*<10^−3^). A visualization of expression ratios by heat map (Fig. 1a) or volcano plot (Fig. 1b) reveals the widespread extent of MYC-dependent regulation of the non-coding transcriptome. We also observed increased levels of total RNA and nuclear RNA per cell at high levels of MYC (Fig. 2). This is in accord with the MYC-driven transcriptional amplification effect reported in previous studies [10, 11].

### MYC is present at the promoters of ncRNA transcripts

**Figure 3:**
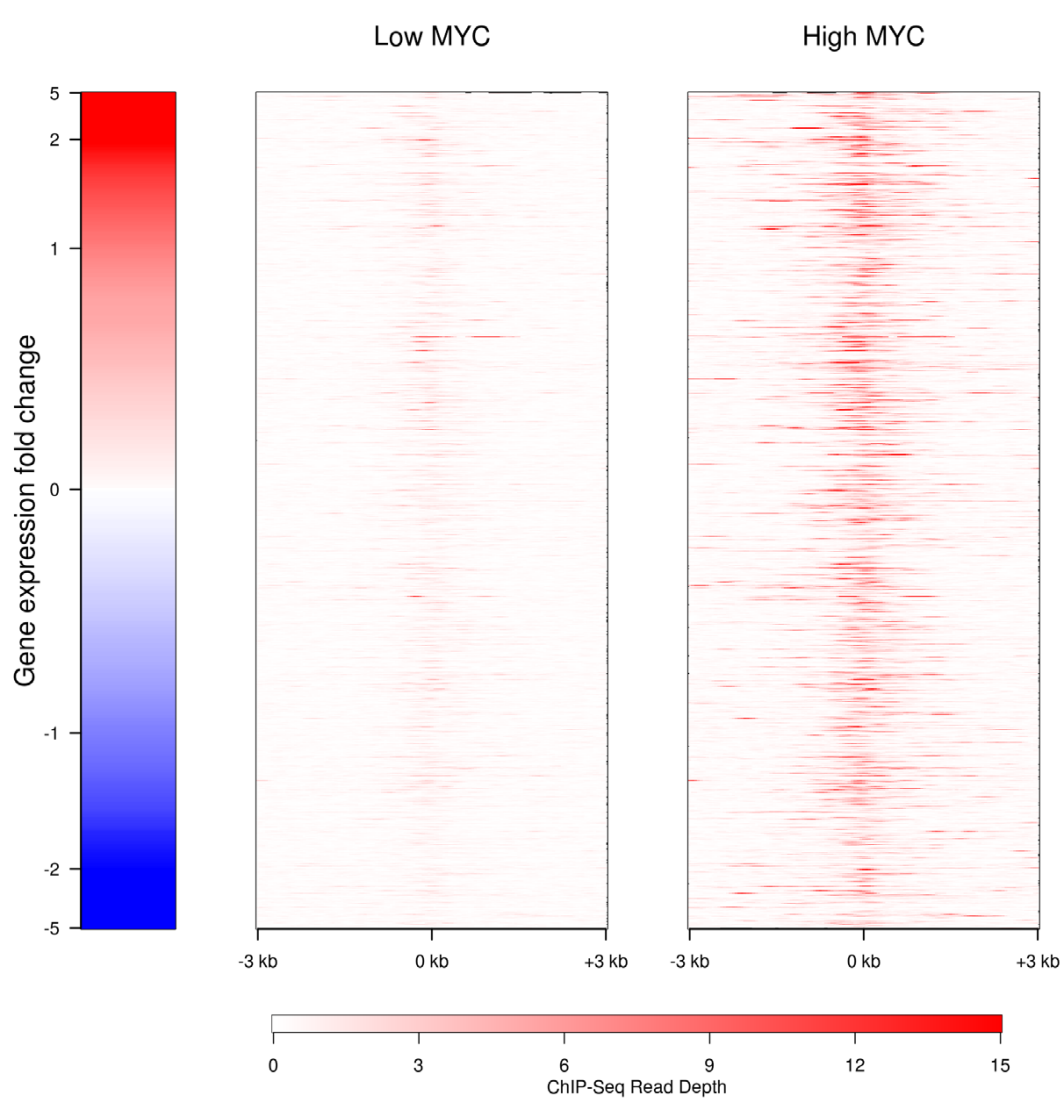
Binding of MYC to the promoters of lncRNAs in the presence of high (right) and low (left) levels of MYC The MYC binding to the proximal promoters of the lncRNAs with detectable transcription is shown in red. Genes are ranked according to their level of expression.

MYC could influence the expression of lncRNA genes either by directly binding to their promoters or by regulating the expression of other transcription factors which subsequently target the non-coding genes. To test for direct regulation by MYC, we have interrogated publicly available P493-6 ChIP-seq (chromatin immunoprecipitation sequencing) data for MYC enrichment proximal to the transcription start sites (TSS) of the 1,273 detected lncRNA genes (Fig. 3). Some MYC-responsive lncRNAs show an increase in MYC occupancy near their TSS similar to that seen for coding transcripts, and MYC typically binds within 1 kb of the TSS. MYC directly binds the TSS in 616 out of 1,273 lncRNAs (48%); this is similar to the situation for coding genes where 6,911 of 10,382 (67%) have MYC present at the TSS. Notably, MYC binding to near TSS was not always associated with a detectable change of expression. This fact may reflect the global effect of MYC on RNA synthesis. These data suggest that lncRNA genes are direct targets of MYC.

### MYC regulates lncRNA transcripts in other cell lines

**Figure 4:**
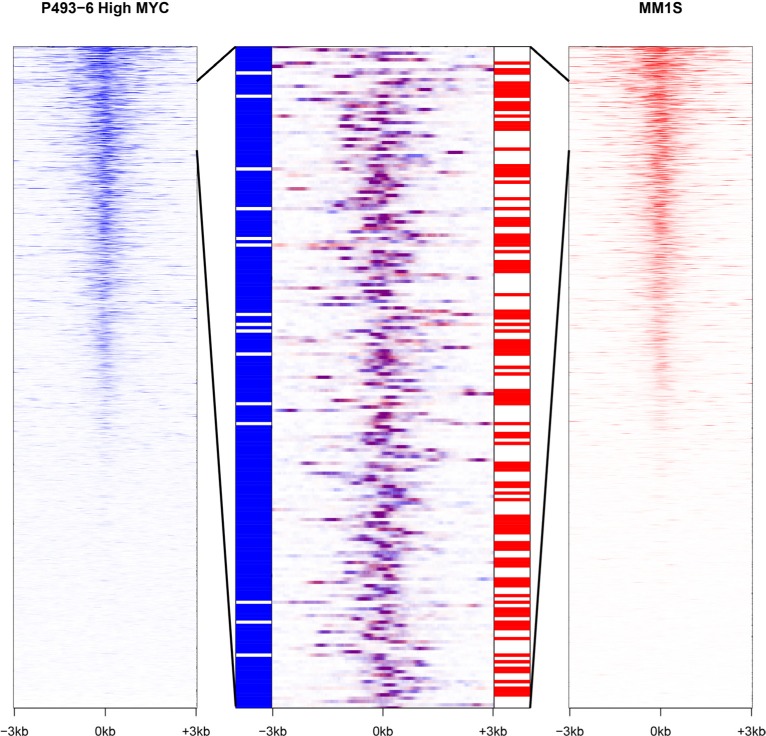
MYC stimulates the transcription of distinct sets of lncRNAs in different cell lines In the extreme right and left panels, lncRNA promoters are ranked according to MYC occupancy for P493-6 cells (high MYC levels) and MM.1S cells respectively. The central panel depicts an overlap of an enlarged segment from the side panels and shows that MYC promoter occupancy is virtually the same in both cell lines (purple color). However, the two cell lines differ starkly in the expression of the corresponding lncRNAs represented in the bar strips flanking the central panel (with blue signifying expression in P493-6 cells and red indicating expression in MM.1S cells).

Public ChIP-seq and RNA-seq datasets are also available for two additional cell lines that show varying levels of MYC expression. The multiple myeloma cell line MM.1S overexpresses MYC as a consequence of a translocation [53], whereas the U87-MG glioblastoma cell line exhibits a more modest increase in MYC expression [54]. The number of promoters bound by MYC is correlated with the amount of MYC present in the cell line. MYC binding is observed at a very similar subset of promoters across all three cell lines. However, at high cellular concentrations of MYC more promoters are targeted than at low levels of MYC (Fig. S1). Yet despite this concordant pattern of binding, each cell line expresses a distinct set of lncRNAs (Fig. 4). Only a minority of lncRNAs are expressed in all three cell lines (14%, 390/2,755). In contrast, a majority of protein-coding genes are expressed in the three cell lines (65%, 8,830/13,650), with fewer transcripts specific to an individual cell line. This observation suggests the existence of additional, possibly differentiation-specific factors that control the transcriptional regulator activity of promoter-bound MYC.

### Classification of MYC-regulated lncRNA transcripts

**Figure 5:**
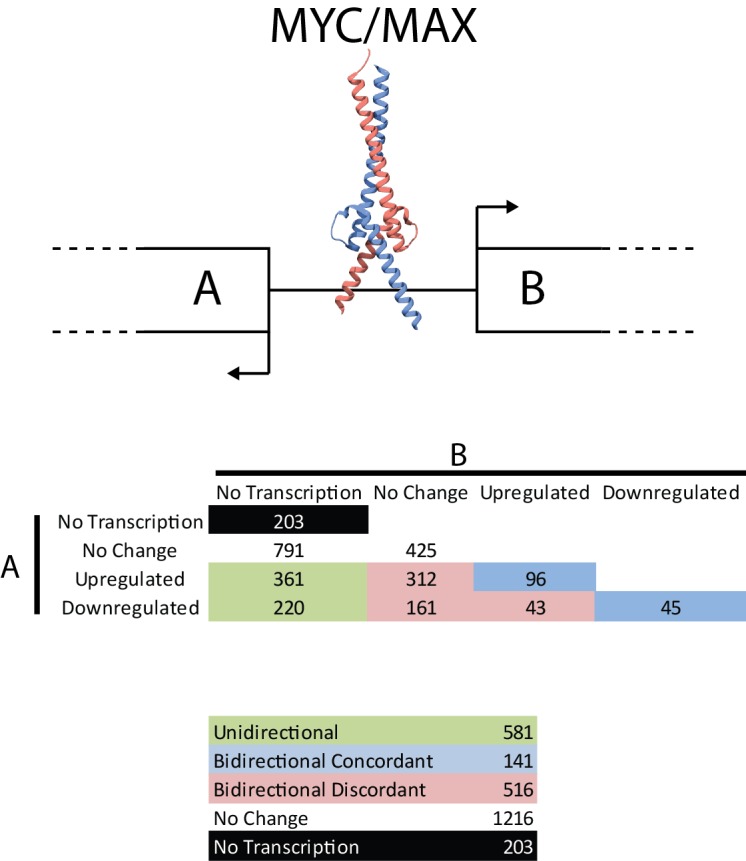
Transcriptional activity from bidirectional promoters MYC binds to bidirectional promoters and drives transcription. ncRNAs are frequently produced from bidirectional promoters. Shown here are the number of MYC-bound bidirectional promoters producing 0 (no transcription), 1 (unidirectional) or 2 (bidirectional) transcripts and whether MYC regulates the genes involved (red, blue and green colored are MYC-regulated, black and white are not MYC-regulated). Despite the symmetric nature of the E-box motif, MYC frequently acts asymmetrically at bidirectional promoters. Numbers indicate the number of bidirectional promoters acting in a specific way.

lncRNAs are frequently produced by bidirectional transcription [24, 55]. There are 2,747 bidirectional promoters which are bound by MYC, and 444 of these involve a lncRNA transcript. The MYC-bound bidirectional promoters were divided into functional categories: bidirectional (both genes expressed) and unidirectional (one of the two genes expressed) and classified according to the impact of high MYC levels upon expression of their respective transcripts (Fig. 5). Many of the bidirectional promoters behave in a discordant manner (19%, 516/2,747) showing a significant preference for one direction over the other. Concordant regulation of both transcripts is seen in the minority of the bidirectional promoters (5%, 141/2,747).

### Polyadenylation of lncRNAs

Whereas the vast majority of coding genes are polyadenylated, many lncRNAs are not [56-59]. To investigate the polyadenylation status of the lncRNA transcriptome, we compared our ribosomal RNA-depleted RNA-seq data with published poly-A-enriched sequencing data prepared from the same cells under identical conditions [13]. A linear relationship was observed between read counts for ribosomal RNA-depleted RNA and poly-A experiments for coding genes (R^2^=0.76, *P*<10^−16^ for low MYC and R^2^=0.58, *P*<10^−16^ for high MYC). Using a linear discriminator classifier, we found only a small number of coding transcripts that lack polyadenylation (0.6%, 69/11,154). Performing a similar analysis for the lncRNA transcripts, we found a weaker, but significant, linear relationship between ribosome-depleted and poly-A methods (low MYC R^2^=0.44, *P*<10^−16^; high MYC R^2^=0.36, *P*<10^−16^). Applying the discriminator established with coding transcripts, a much larger number of non-coding transcripts (18%, 241/1,371) were found to lack polyadenylation.

### Validation of RNA-seq data

**Figure 6:**
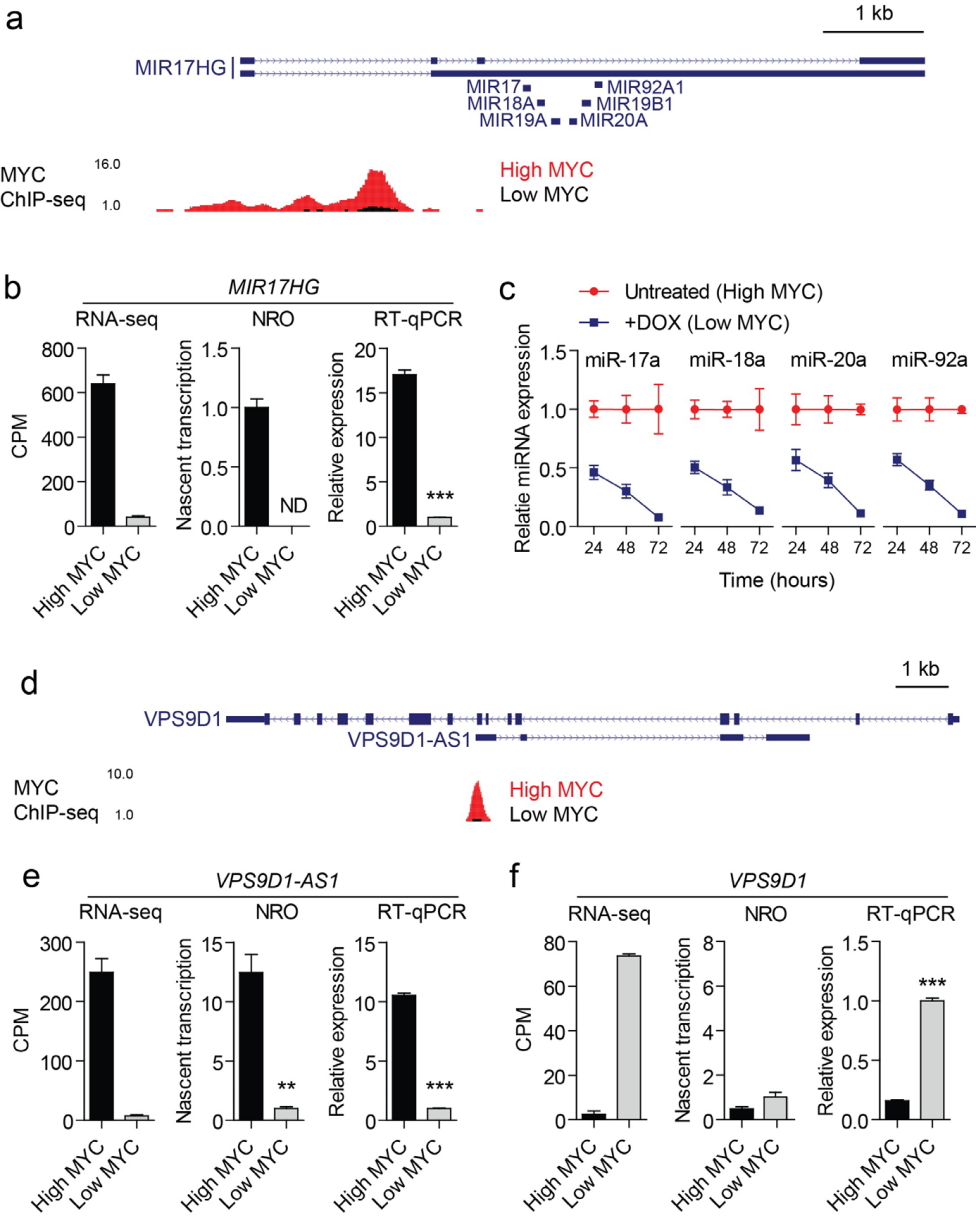
Validation data for two representative MYC-regulated lncRNAs (a) MYC ChIP-seq data were visualized in the UCSC genome browser for the *MIR17HG* locus. The locations of miRNA precursors are indicated. (b) Expression of *MIR17HG* in high MYC and low MYC conditions was determined by RNA-seq, NRO and steady-state RT-qPCR. (c) Mature miRNAs from the miR-17-92 cluster were also affected by MYC levels. Addition of doxycycline (DOX) reduced miRNA levels. This effect of the repression increased over 72 hours. (d) MYC ChIP-seq data were visualized at the *VPS9D1-AS1* locus. The *VPS9D1-AS1* gene is antisense to, and resides completely within, its corresponding sense coding gene *VPS9D1*. Expression of (e) *VPS9D1-AS1* and (f) *VPS9D1* were determined by RNA-seq, NRO and RT-qPCR. This sense-antisense pair was discordantly regulated such that *VPS9D1-AS1* was transcriptionally upregulated under high MYC expression, whereas *VPS9D1* was correspondingly downregulated suggesting possible regulatory relationship. The change in *VPS9D1* expression observed by RNA-seq and steady-state RT-qPCR was not recapitulated in the NRO data indicating that *VPS9D1* is regulated at the post-transcriptional level. Values are mean +/− SEM, *n*=3, **P<0.01, ***P<0.001, ND not detected.

To validate the RNA-seq data, we selected eight representative lncRNAs (Table 1) based on the following criteria: (1) significantly upregulated by MYC by >8 fold in the RNA-seq dataset, (2) well annotated in UCSC (University of California Santa Cruz) genome browser, (3) detected with >5 reads per sample, and (4) presence of a MYC binding ChIP-seq peak proximal (+/− 0.5kb) to the lncRNA TSS. These eight lncRNAs could be grouped into three general classes: (1) host genes for small RNAs (*DANCR*, *MIR17HG*, *SNHG15*, *SNHG16*), (2) concordantly regulated sense-antisense pairs (*MCM3AP-AS1*, *USP2-AS1*), and (3) discordantly regulated sense-antisense pairs (*KTN1-AS1*, *VPS9D1-AS1*). Analysis of exon-exon junction spanning split reads supported the intron-exon gene models annotated in the UCSC genome browser hg19 for all eight lncRNA genes. Parallel nuclear run-on (NRO) and steady-state gene expression analysis by RT-qPCR (quantitative reverse transcription PCR) were performed on P493-6 cultures in the presence and absence of doxycycline. Given the precedent for sense gene regulation by proximal antisense transcripts, we also investigated expression of neighboring or overlapping transcripts where applicable. Using this strategy, RNA-seq expression data were validated at both the transcriptional and steady-state level for all eight lncRNAs. A detailed example of two of these validated genes is presented in Fig. 6; the data on the remaining 6 validated lncRNAs are shown in Figs. S2 through S7. A positive correlation (Pearson r=0.927, *P*=0.0027) was observed between NRO and RT-qPCR expression ratios indicating that these lncRNAs are predominantly regulated at the level of transcription.

**Table 1:** Representative MYC-regulated lncRNAs Eight putative MYC-regulated lncRNAs were selected for validation studies. CPM, counts per million.

lncRNA (RefSeq ID)	Fold up-regulated RNA-seq (RT-qPCR)	Mean CPM	Nuclear/Cytoplasmic	Poly-A	t_1/2_ (minutes)	Predicted function
*DANCR* (NR_024031.1)	+13.3 (+9.4)	48	Cytoplasmic	+	132	Small RNA host gene/Unknown cytoplasmic function
*KTN1-AS1* (NR_027123.1)	+21.9 (+12.5)	6.6	Both	+	66	Discordant bidirectional promoter (*KTN1*)
MCM3AP-AS1 (NR_002776.4)	+8.5 (+1.7)	8.9	Nuclear	+	57	Concordant bidirectional promoter (*LSS*)
*MIR17HG* (NR_027349.1)	+15.5 (+17.0)	24	Nuclear	+	25	Small RNA host gene
*SNHG15* (NR_003697.1)	+9.0 (+3.0)	23	Both	+	31	Small RNA host gene
*SNHG16* (NR_038111.1)	+14.5 (+10.4)	69	Cytoplasmic	+	125	Small RNA host gene
*VPS9D1-AS1* (NR_036480.1)	+33.3 (+10.5)	8.0	Both	+	107	Post-transcriptional regulator of *VPS9D1*
*USP2-AS1* (NR_034160.1)	+21.9 (+21.2)	22	Both	+	127	Discordant bidirectional promoter (*USP2)*

The properties of lncRNAs can provide clues as to their function. For example, lncRNAs which execute their functions at the transcriptional level are expected to be enriched in the nucleus. To investigate the sub-cellular localization of the eight representative lncRNAs, P493-6 cells were separated into nuclear and cytoplasmic fractions and lncRNA levels assessed by RT-qPCR. Two transcripts were predominantly cytoplasmic (*DANCR* and *SNHG16*) whereas three transcripts were enriched in the nucleus (*MCM3AP-AS1*, *MIR17HG* and *USP2-AS1*). The remaining transcripts were evenly distributed between nucleus and cytoplasm (Fig. S8). Interestingly, a positive correlation (Pearson r=0.675, *P*=0.023) was observed between transcript abundance and the cytoplasmic:nuclear distribution ratio such that the more abundant lncRNAs tended to be enriched in the cytoplasm. All eight of the validated lncRNAs could be amplified from oligo dT primed cDNA libraries suggesting that these transcripts are polyadenylated, and that polyadenylation is unlikely to be a determinant of sub-cellular localization for this subset of transcripts.

Determination of transcript stability can be informative with respect to function as one class of lncRNAs (the PROMPTs or PROMoter uPstream Transcripts) is known to be highly unstable [60]. Equally, non-functional transcripts which arise as a result of ‘noisy transcription’ might also be expected to be short-lived. To investigate the transcript half-lives of the eight representative lncRNAs, P493-6 cultures were treated with Actinomycin D in order to inhibit transcription [61], RNA was collected over a series of time points, transcript levels were measured by PCR, and transcript half-lives (t_1/2_) calculated (Fig. S9, Table 1). The half-lives of *MYC* (fast turnover: t_1/2_=30 minutes) and *GAPDH* (slow turnover: t_1/2_=215 minutes) were also measured for comparison. The eight lncRNAs showed transcript stabilities that extended from half-lives of 132 minutes (*DANCR*) to 25 minutes (*MIR17HG*). These results demonstrate that the eight validated lncRNAs have mRNA-like stability properties and are unlikely to be PROMPTs (despite some being promoter-overlapping) or a consequence of transcriptional noise.

## DISCUSSION

Recent work has suggested that MYC acts as an amplifier of global transcriptional output [10, 11]. The present study shows that in addition to affecting the coding transcriptome, overexpression of MYC also induces widespread changes in non-coding transcription. MYC binds to the promoters of lncRNA transcripts, but that interaction is not always sufficient to modulate transcription of the target promoter. The general picture that emerges from the data is that the extent and degree of MYC-mediated transcriptional activity is similar for non-coding and coding transcripts. However, there is one significant difference. Expression from MYC-binding lncRNA promoters is, in contrast to the observations on coding genes, largely distinct in different cell types. There are cell type-specific subsets of lncRNAs. This phenomenon has been documented previously. An investigation of the transcriptional landscape of multiple human cell lines found that 29% of lncRNAs are expressed specifically in a single cell type, whereas only 10% are expressed in all cell types [62]. This specificity could reflect the regulatory functions of many lncRNAs and suggests the existence of additional, possibly differentiation-specific factors that control the effectiveness of promoter-bound MYC.

The functions of most lncRNAs are currently unknown. There are, however, broad criteria that define various categories of lncRNAs and that are relevant to function. These include antisense RNAs likely to control their sense counterparts and nuclear versus cytoplasmic localization that could serve as broad indicator of function. Transcriptional activities initiated from bidirectional promoters can be divided into concordant and discordant with implied consequences on function.

Eight lncRNAs that were upregulated in high MYC expression conditions were validated by RT-qPCR. MYC binding was enriched at the promoters of these genes when MYC expression was high, and regulation at the transcriptional level confirmed by NRO. This set of representative MYC-regulated lncRNAs exhibited diverse properties in terms of their genomic organization, sub-cellular localization, abundance, and transcript stability (Table 1) although all eight lncRNAs were found to be polyadenylated. Some functions of the eight validated lncRNAs are apparent. *DANCR*, *MIR17HG*, *SNHG15* and *SNHG16* act as precursors of small RNA species including miRNAs and snoRNAs. These function in post-transcriptional gene regulation and rRNA editing/maturation respectively.

The oncogenic activities of MYC are likely to include regulation of lncRNAs. The transcriptional activation of negative regulators could be a major mechanism of MYC-mediated gene silencing. Among the eight validated lncRNAs, there are three with relevance to cancer, *MIR17HG*, *USP2-AS1*, and *DANCR;* the roles of the remaining five are currently not known. *MIR17HG* encodes the miR-17-92 cluster. This miRNA cluster is upregulated by MYC (Fig. 6), and we have confirmed this upregulation for the processed, mature miRNAs. Their roles in cancer have been widely documented [16, 63-66]. The transcription of *USP2-AS1* is also activated by MYC. The corresponding sense gene *USP2* is a ubiquitin-specific cysteine protease that shows diverse pro-oncogenic activities [67] and could be controlled by a regulatory circuit that includes *USP2-AS1*. *DANCR* is also upregulated by MYC. It generates miR-4449 and *SNORA26* and is downregulated during differentiation [68, 69], hence upregulated *DANCR* might contribute to cancer by maintaining a pro-proliferative state.

The regulatory effect of MYC on a broad segment of the non-coding transcriptome opens up a new area of MYC activity. The challenge is now to identify the cancer-relevant lncRNA targets of MYC and determine their functions.

## MATERIALS AND METHODS

### Cell Culture

P493-6 cells suspensions were maintained in RPMI 1640 (Life Technologies) containing 10% tetracycline-free FBS (Gemini) and supplemented with 2 mM L-glutamine, 100 U penicillin, 0.1 mg/mL streptomycin (Sigma-Aldrich) at 37°C and 5% CO_2_ at cell densities between 10^5^ and 10^6^ cells/mL. To inhibit MYC expression, doxycycline (Sigma-Aldrich) was added to the media to a final concentration of 0.1 μg/mL.

### Bioinformatics

Both ribosome-depleted and poly-A-enriched RNA-seq +/− doxycycline P493-6 were previously published and were obtained from NCBI SRA accession numbers: SRR1313741, SRR1313742, SRR1313743, SRR1313735, SRR1313736, SRR1313737, SRR567561, SRR567562 [13, 70]. U87MG (SRR492058, SRR492059) [23] and MM.1S (SRR931814) [71] were also obtained through NCBI SRA. Reads were mapped using the RNA STAR aligner (2 pass method) [72] to hg19. Counts aligning to Gencode v19 coding and non-coding genes [50, 51]were obtained using HTSeq-count [50, 52]. Differential expression was analyzed using EdgeR. Coverage data was obtained using Bedtools genomecov [73] and UCSC Kent utilities [74].

ChIP-seq data for P493-6, MM.1S and U87 were previously published [11] and were obtained through NCBI SRA accession numbers: SRR444481, SRR444479, SRR444467, SRR444466, SRR444464, SRR444432, SRR444430, SRR444429. The raw reads were mapped to hg19 using BWA [75, 76]. Binding peaks for MYC were determined using MACS [77] and annotated using Homer [78]. Coverage plots were generated from Homer annotation data and plotted in R.

Poly-A and rRNA-depleted RNA-seq data were compared. Raw counts were normalized to counts per million and log_2_ transformed. Genes with less than 5 counts in the lowest sample were filtered out. Known polyadenylated transcripts and those lacking polyadenylation [57] were used to construct a training set for linear discriminant analysis.

### Nuclear Run-On

Treated P493-6 cultures were counted using a Countess Automated Cell Counter (Life Technologies) and 3.6×10^6^ cells were aliquoted per NRO sample. 5% of the cells were removed and stored separately for parallel steady-state RT-qPCR analysis. Nuclei were harvested in NP-40 Lysis Buffer (10 mM Tris-HCl pH 7.4, 10 mM NaCl, 3 mM MgCl_2_, 0.5% NP-40) and resuspended in Nuclei Storage Buffer (50 mM Tris-HCl pH 8.3, 0.1 mM EDTA, 5 mM MgCl_2_, 40% glycerol). NRO transcription was performed by adding 2x transcription buffer (20 mM Tris-HCl pH 8.3, 5 mM MgCl_2_, 300 mM KCl, 4 mM DTT) supplemented with 1 mM ATP, 1 mM CTP, 1 mM GTP, 0.5 mM UTP, 0.5 mM BrUTP and 100 U RNaseOUT and incubating for 30 minutes at 30°C. A spike-in control RNA (Renilla luciferase mRNA prepared by T7 *in vitro* transcription in the presence of BrUTP) was added to act as an external reference for data normalization that was independent of global changes in nuclear RNA content. NRO reactions were terminated by immediately lysing the nuclei and extracting RNA using the Maxwell-16 System RNA LEV kit (Promega). Genomic DNA was removed by a DNase I treatment step included in the extraction protocol. ~500 ng of NRO-RNA per sample were immunoprecipitated using mouse monoclonal anti-BrdU antibodies (Santa Cruz Biotechnology) and Protein G Dynabeads (Life Technologies). Beads were blocked with blocking buffer (PBST, 0.1% Polyvinylpyrrolidone, 0.1% UltraPure BSA) and NRO-RNA samples incubated with the beads for 30 minutes at room temperature on a rotating platform. Subsequently, the beads were washed three times with PBST (supplemented with 8 U/ml RNaseOUT). Immunoprecipitated NRO-RNAs were extracted using TRIzol Reagent (Life Technologies) according to manufacturer's instructions and RT-qPCR performed.

### Cellular Fractionation

P493-6 nuclear and cytoplasmic RNA samples were prepared using the Protein and RNA Isolation System (PARIS) kit (Life Technologies) and genomic DNA removed using the TURBO DNA-*free* kit (Life Technologies) according to manufacturer's instructions.

### Determination of Transcript Half-life

P493-6 cultures were treated with 2 μg/mL Actinomycin D and then harvested at 30, 60, 120 and 240 minutes later. RNA was extracted and transcript levels assessed by RT-qPCR. Transcript half-lives were calculated using the formula:

$$t_{\frac{1}{2}}\;=\;\frac{t\;\ln\;2}{\ln\;N_{0}-\ln\;N}$$

The mean of four different time-points was reported.

### RT-qPCR

RNA samples were reverse-transcribed using the High Capacity cDNA Synthesis kit (Life Technologies) according to manufacturer's instructions (using a random priming or oligo dT-priming strategy as appropriate). qPCR was performed on either a Mastercycler ep RealPlex 2 (Eppendorf) or LightCycler 96 (Roche) real-time PCR instrument. Reactions were prepared using KAPA SYBR FAST qPCR Master Mix (KAPA Biosystems) and universal cycling conditions (95°C for 3 minutes followed by 40 cycles of 95°C for 3 seconds and 60°C for 30 seconds). Reaction specificity was confirmed by melt curve analysis. All primer sequences are listed in Table S2. For miRNA quantification the TaqMan MicroRNA Reverse Transcription Kit (Life Technologies) was used with the following Small RNA TaqMan Assays: miR-17a, miR-18a, miR-20a and miR-92a (#4427975, #000394, #000580 and #000431). Steady-state gene expression measurements were normalized to the geometric mean [79] of *RPLP0* and *RPL10* (two transcripts that were found to be largely unaffected by MYC-driven transcriptional amplification). For NRO-RT-qPCR, data were normalized to a bromouridylated spike-in control transcript (RLuc) added at the NRO phase in order to provide an external reference transcript independent of MYC-induced changes in global transcriptional activity. miRNA data were normalized to RNU6 (#001973) expression.

## SUPPLEMENTARY MATERIAL, FIGURES AND TABLES




