# Supplementary material for: MYC regulates the non-coding transcriptome
Source: Oncotarget. 2014 Dec 30;5(24):12543–54. doi: 10.18632/oncotarget.3033 (PMC4350361; doi:10.18632/oncotarget.3033)
Supplement: Supplementary file 1 [file oncotarget-05-12543-s001.pdf]

MYC regulates the non-coding transcriptome

Supplementary Material

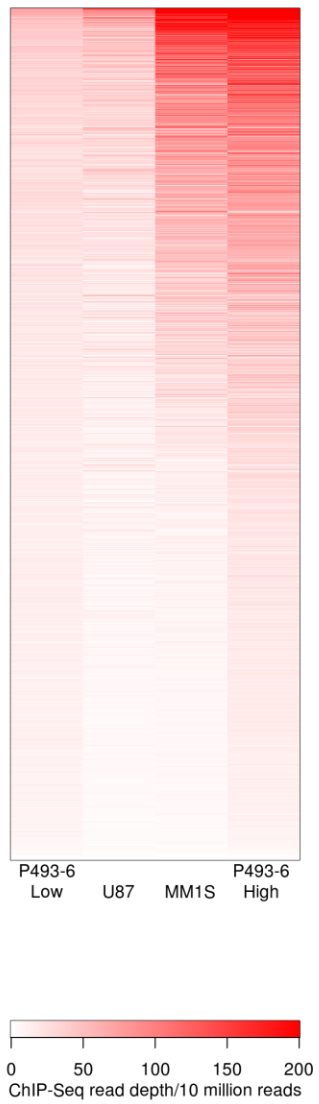

Supplemental Figure 1: MYC binds to non-coding RNA promoters in P493-6, MM.1S and U87 cells. The number of ChIP-seq reads (normalized by the total number of reads) within 3 kb of the promoters was determined for lncRNAs expressed in any of the three cell lines. Columns are arranged by increasing amounts of MYC, and rows are ordered by the average ChIP-seq intensity in the four datasets.

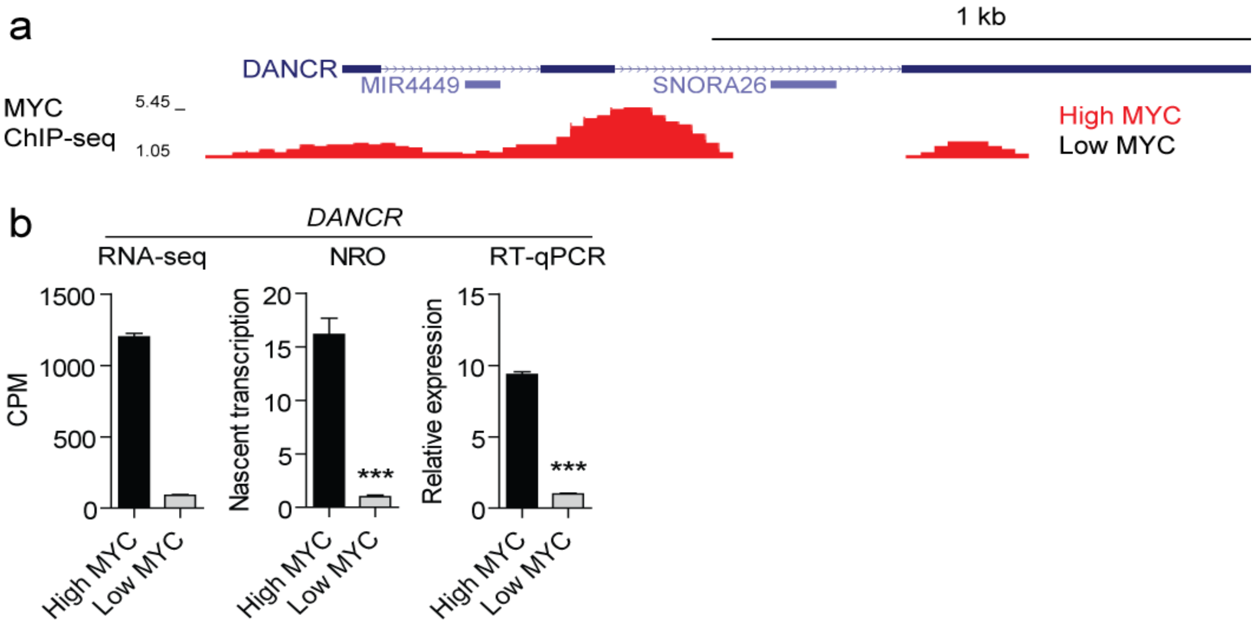

Supplemental Figure 2: Validation data for the MYC-regulated lncRNA *DANCR*. (a) MYC ChIP-seq data were visualized in the UCSC genome browser for the *DANCR* locus. The locations of miRNA precursor/snoRNAs are indicated. (b) Expression of *DANCR* in high MYC and low MYC conditions was determined by RNA-seq, NRO and steady-state RT-qPCR. Values are mean + SEM, n=3, ***P<0.001.

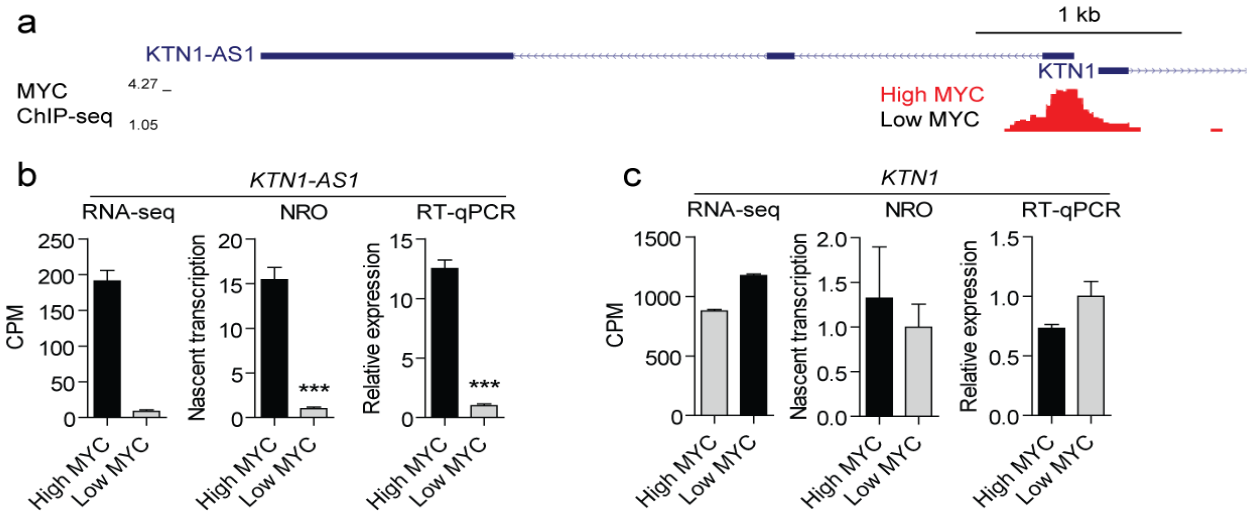

Supplemental Figure 3: Validation data for the MYC-regulated lncRNA *KTN1-AS1*. (a) MYC ChIP-seq data were visualized in the UCSC genome browser for the *KTN1-AS1* locus. *KTN1-AS1* shares a bi-directional promoter with *KTN1*. Expression of (b) *KTN1-AS1* and (c) *KTN1* in high MYC and low MYC conditions was determined by RNA-seq, NRO and steady-state RT-qPCR. Values are mean + SEM, n=3, ***P<0.001.

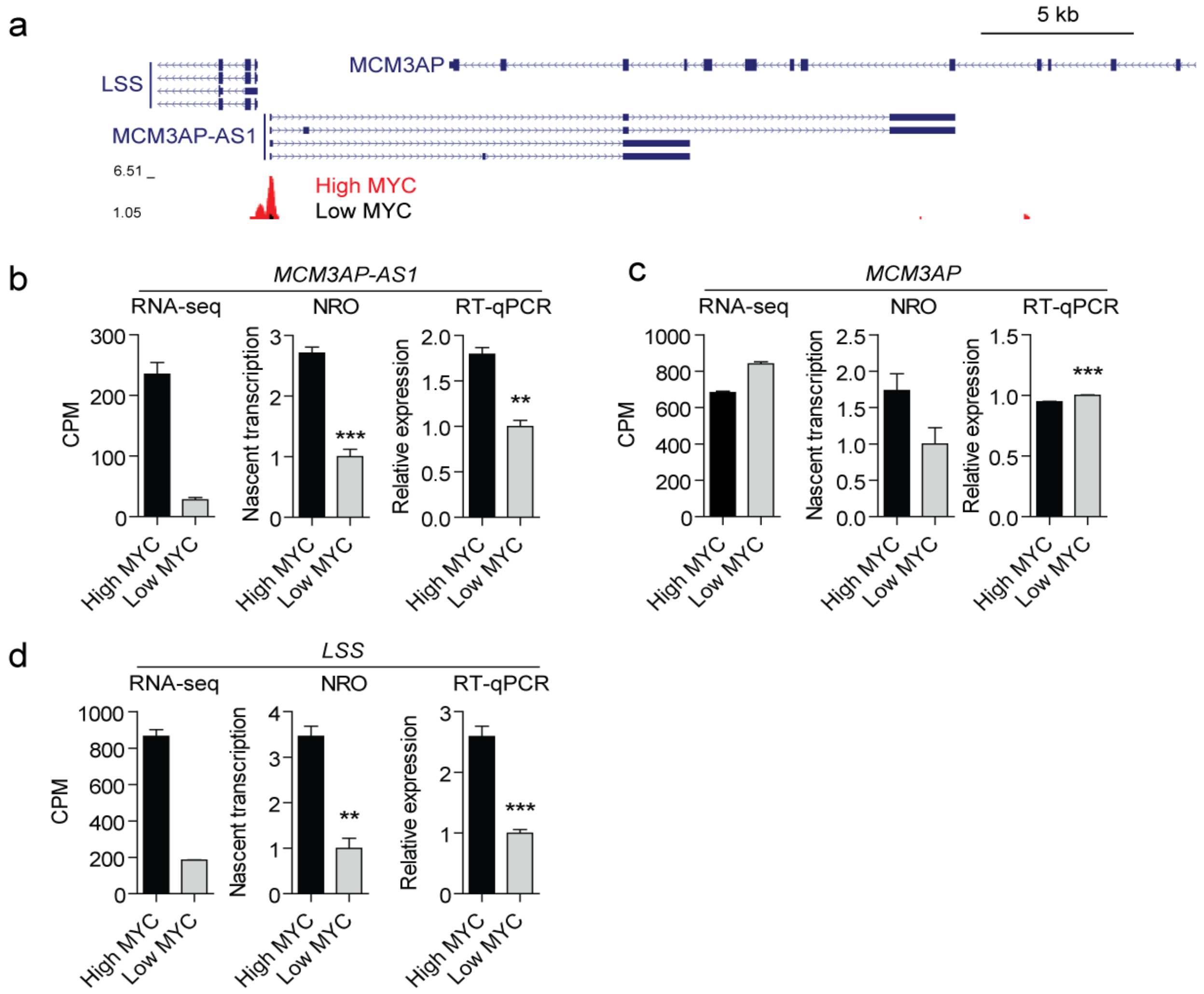

Supplemental Figure 4: Validation data for the MYC-regulated lncRNA *MCM3AP-AS1*. (a) MYC ChIP-seq data were visualized in the UCSC genome browser for the *MCM3AP-AS1* locus. The *MCM3AP-AS1* gene is an antisense lncRNA that overlaps with the 3′ terminus of the protein-coding gene *MCM3AP*. Additionally, *MCM3AP-AS1* is derived from a bidirectional promoter shared with a second protein-coding gene *LSS*. Expression of (b) *MCM3AP-AS1*, (c) *MCM3AP*, and (d) *LSS* in high MYC and low MYC conditions was determined by RNA-seq, NRO and steady-state RT-qPCR. *MCM3AP* was unaffected by MYC expression levels suggesting that there is no regulatory relationship between it and *MCM3AP-AS1*. Conversely, LSS was concordantly up-regulated with *MCM3AP-AS1* suggesting that MYC transcriptionally activates transcription of this promoter in both directions. Values are mean + SEM, n=3, **P<0.01, ***P<0.001.

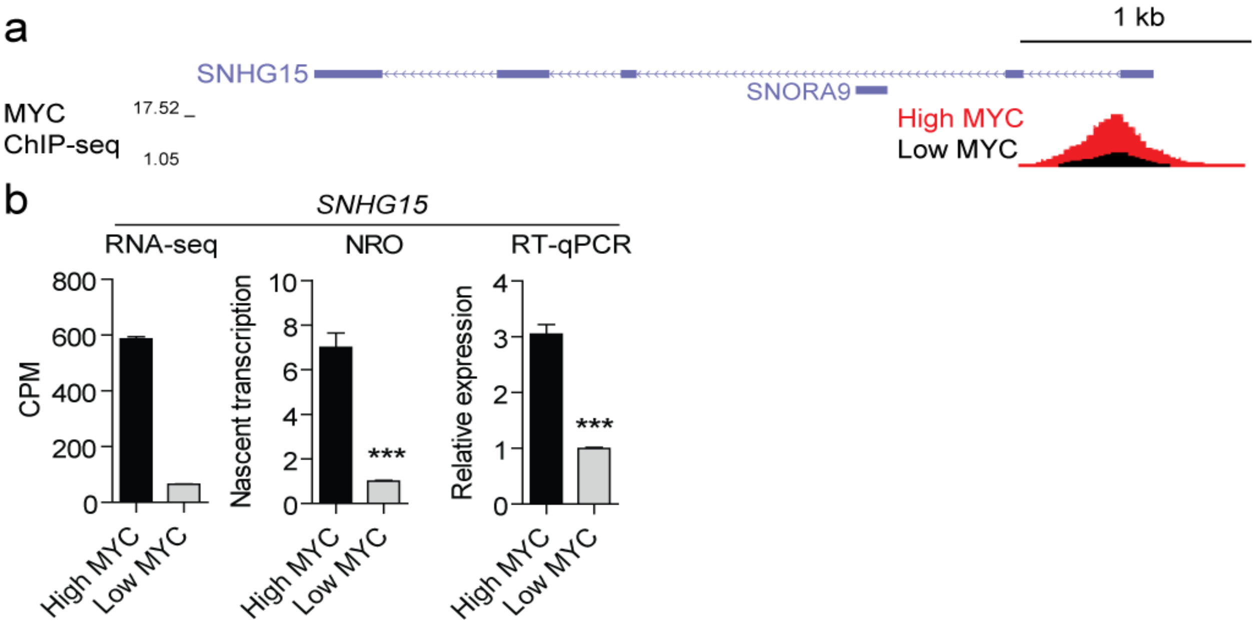

Supplemental Figure 5: Validation data for the MYC-regulated small nucleolar (sno) RNA host gene *SNHG15*. (a) MYC ChIP-seq data were visualized in the UCSC genome browser for the *SNHG15* locus. Locations of snoRNAs are indicated. (b) Expression of *SNHG15* in high MYC and low MYC conditions was determined by RNA-seq, NRO and steady-state RT-qPCR. Values are mean + SEM, n=3, ***P<0.001.

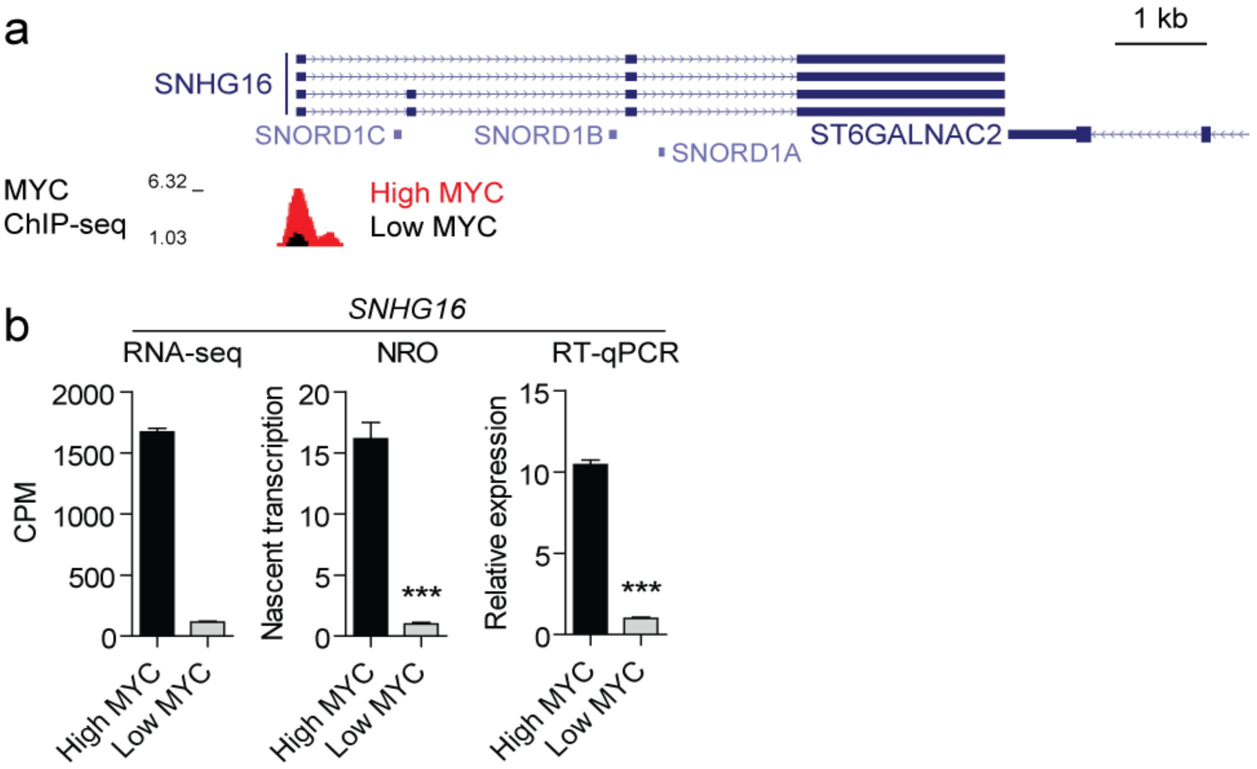

Supplemental Figure 6: Validation data for the MYC-regulated snoRNA host gene *SNHG16*. (a) MYC ChIP-seq data were visualized in the UCSC genome browser for the *SNHG16* locus. Locations of snoRNAs are indicated. (b) Expression of *SNHG16* in high MYC and low MYC conditions was determined by RNA-seq, NRO and steady-state RT-qPCR. Values are mean + SEM, n=3, ***P<0.001.

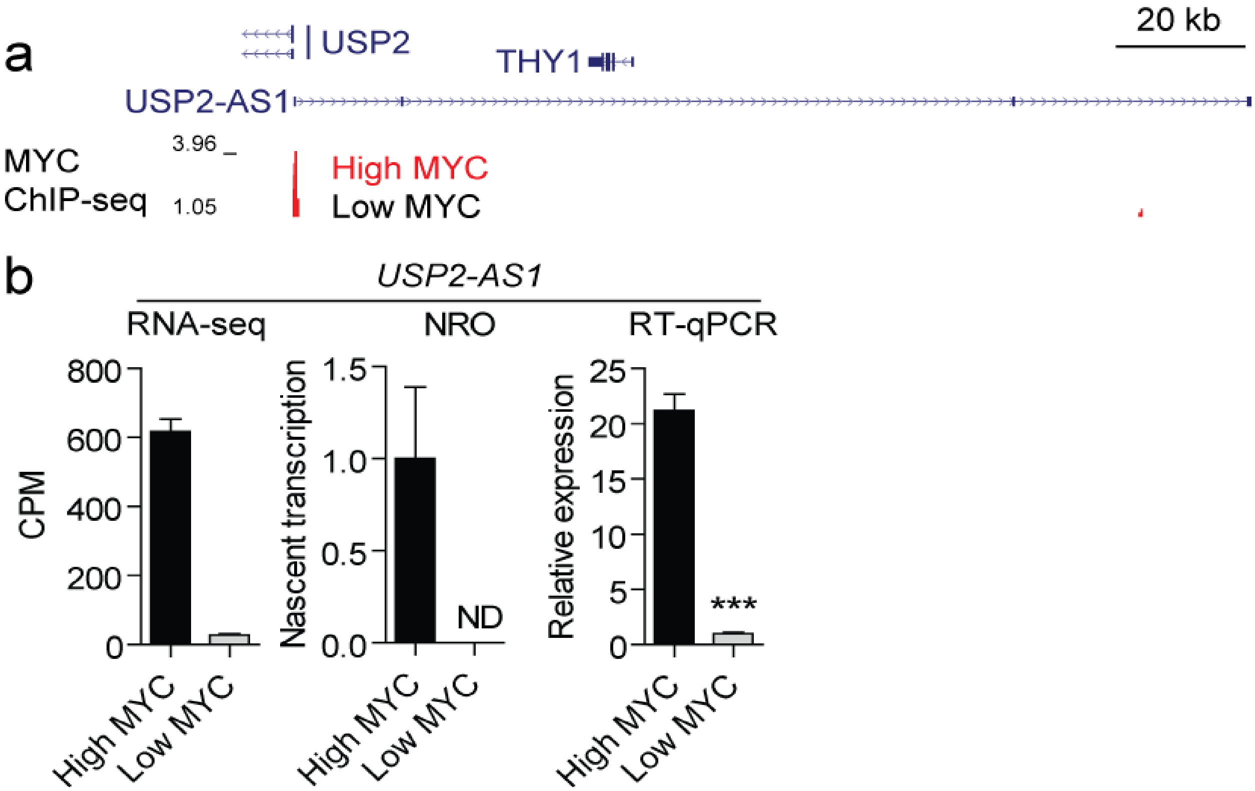

Supplemental Figure 7: Validation data for the MYC-regulated lncRNA *USP2-AS1*. (a) MYC ChIP-seq data were visualized in the UCSC genome browser for the *MCM3AP-AS1* locus. (b) Expression of *USP2-AS1* in high MYC and low MYC conditions was determined by RNA-seq, NRO and steady-state RT-qPCR. Values are mean + SEM, n=3, ***P<0.001, ND not detected.

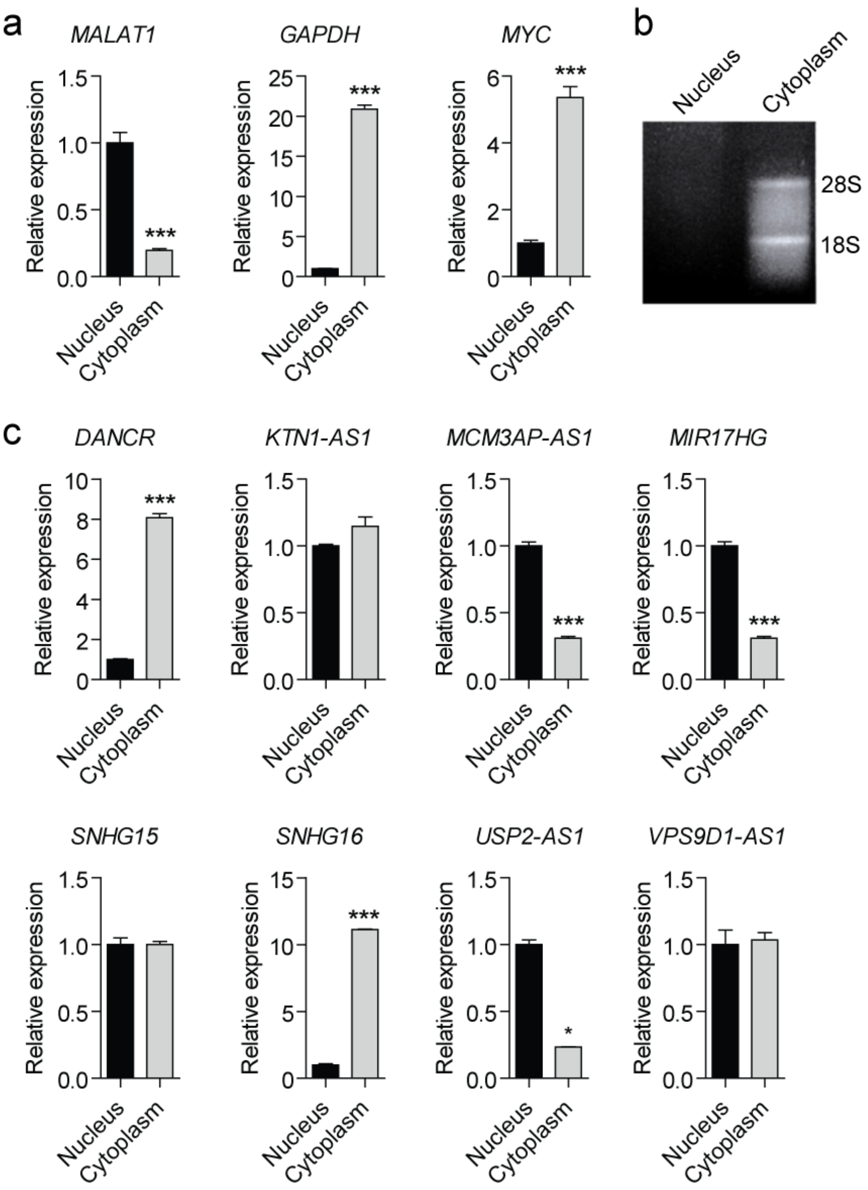

Supplemental Figure 8: Nucleocytoplasmic distribution of lncRNAs. P493-6 cultures were separated into nuclear and cytoplasmic fractions. (a) Cellular fractionation was confirmed by measuring the transcript abundance of *MALAT1* (nuclear) or *GAPDH* and *MYC* (cytoplasmic) in either fraction. (b) Total RNA from both fractions was separated by agarose gel electrophoresis. 18S and 28S ribosomal RNAs were observed in the cytoplasmic fraction only. (c) The relative expression of the validated lncRNAs was determined in the nucleus and cytoplasm. All values are mean + SEM, n=3, *P<0.05, ***P<0.001.

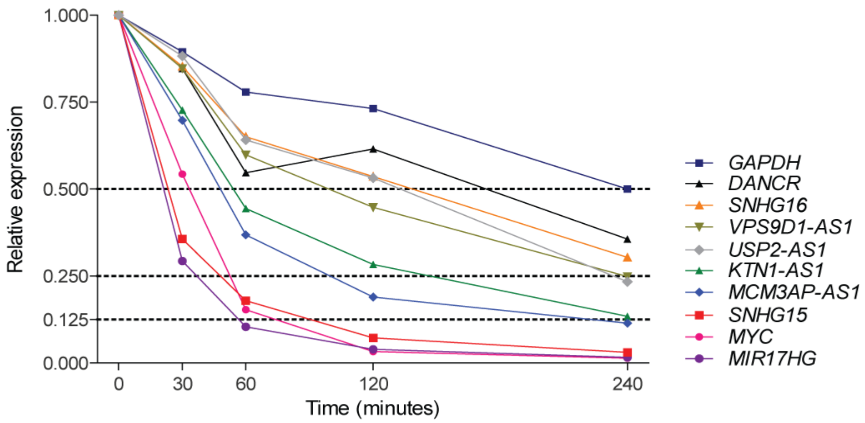

Supplemental Figure 9: Transcript stability of lncRNAs. P493-6 cultures were treated with Actinomycin D and cultures harvested at a series of time points later. RNA was extracted, RT-qPCR performed and transcript half-times ($t_{1/2}$) determined as described in Materials and Methods. Transcript decay curves are shown for the representative lncRNAs with *GAPDH* and *MYC* included for comparison.

Supplemental Table 1: A census of lncRNAs expressed in P493-6 cells and presented as counts per million with high MYC and low MYC as regulated by 0.1 ug/mL doxycycline at 24 hours. Average log_2(fold change) as well as significance values are included.

Supplemental Table 2: List of RT-qPCR primers used in this study. Note that primer sets were designed to be suitable for both steady-state gene expression analysis and nuclear run-on.

	FWD	REV
**RLuc**	GTAACGCTGCCTCCAGCTAC	CCAAGCGGTGAGGTACTTGT
**MYC**	GCAAAAGCTCATTTCTGAAGAGG	TTCATAGGTGATTGCTCAGGAC
**GAPDH**	CGCTGAGTACGTCGTGGAGTC	GCAGGAGGCATTGCTGATGA
**MALAT1**	GCGTAATGGAAAGTAAAGCCC	CAAACACCTCACAAAACCCC
**RPLP0**	GAAACTCTGCATTCTCGCTTC	GGTGTAATCCGTCTCCACAG
**RPL10**	CCTCTTTCCCTTCGGTGTG	AATCTTGGCATCAGGGACAC
**DANCR**	GCTGACCCTTACCCTGAATAC	CTTCGGTGTAGCAAGTCTGG
**KTN1-AS1**	CGTGAAAGGTGGGATCTGTAG	GAACTGCCTGTCTCGTATAGC
**MCM3AP-AS1**	CACCTCTTGCTCACTCTGATG	TCTACTTCCCAGCTCACTCTAG
**MIR17HG**	GACCTGTCTAACTACAAGCCAG	TGTTCTCCAGGAAGTTGCAG
**SNHG15**	CCTTGGCACCTTAATTGAGC	ATTCTGGAAGCAGAGAACCTG
**SNHG16**	CTGCTATCATAGAGACCAAGGAG	TTACTGGCACGAGGACAAAG
**USP2-AS1**	ACCAAATCCCCAGACCAATG	AGCGAGATGAAGAGTTTACACC
**VPS9D1-AS1**	ACTCCCATTTTCTCATCCCG	GGAGAGCAAAGTCCCAGAAG
**KTN1**	TCTGAGAGTGTACCTCGAGAC	GCACAGGCTTTTGTTTCTTTTC
**LSS**	AGGTAGGTCCACGTCTGC	GCCCCTACAAGACCGAG
**MCM3AP**	GTCTCCTTTGCAGTCTGGAAG	TTGCTGTGAGGATGTCTGTG
**VPS9D1**	CTCAGACGTCCCCAGGAACTG	GCCAGACAAGGACAGCTCGTTC
